# Relationship between radiological severity and physical and mental health in elderly individuals with knee osteoarthritis

**DOI:** 10.1186/s13075-020-02280-2

**Published:** 2020-08-12

**Authors:** Isadora Cristina Ribeiro, Arlete Maria Valente Coimbra, Beatriz Lavras Costallat, Ibsen Bellini Coimbra

**Affiliations:** Department of Rheumatology, Faculty of Medical Sciences, State University of Campinas, Rua Tessália Vieira de Camargo, 126 Cidade Universitária Zeferino Vaz, Campinas, São Paulo CEP 13083-887 Brazil

**Keywords:** Radiography, Osteoarthritis, Elderly

## Abstract

**Background:**

This study aimed to investigate the relationship between radiological severity, as assessed by the individual grades and grouped grades (grades “0 and 1” and “2 to 4”) of the Kellgren-Lawrence scale (K&Ls), and depression symptoms, cognitive loss, risk of falls, and quality of life in relation to knee osteoarthritis, as assessed by other instruments.

**Methods:**

Data recorded between 2013 and 2014 in Amparo (São Paulo, Brazil) were retrieved for analysis. A total of 181 elderly patients who had knee osteoarthritis and underwent a radiologic exam were evaluated for depressive symptoms, cognitive loss, quality of life, and risk of falls by the Geriatric Depression Scale (GDS), Mini-Mental State Examination (MMSE), Western Ontario and McMaster Universities Osteoarthritis Index (WOMAC), timed up and go test (TUG), and Berg balance scale (BBS). For statistical analyses, Fisher’s exact test, Mann-Whitney test, Kruskal-Wallis test, and Spearman’s coefficient analysis were used.

**Results:**

There was no significant relationship between the scores of the instruments investigated and the individual K&Ls grades. However, when the K&Ls scores were assessed by groups, grades “2 to 4” were associated with the worst WOMAC score and the highest frequency and risk of falls according to the BBS but not according to TUG. For the GDS and MMSE, no significant relationships with the K&Ls grades were found. In addition, the K&Ls grade was correlated with the WOMAC score, regardless of the domain.

**Conclusion:**

The radiological scores of the Kellgren-Lawrence (K&L) scale were associated with poorer WOMAC and BBS scores only when the K&Ls scores were evaluated in groups, and the WOMAC score was associated with an increase in the radiological grade.

## Introduction

Knee osteoarthritis (KOA) is a chronic, low-grade, inflammatory form of arthritis that affects all joint structures, such as the hyaline cartilage, synovial membrane, and subchondral bone, as well as other joint tissues; thus, it is considered one of the main causes of physical disability in elderly individuals. The development and progression of KOA can lead to joint impairment, followed by creaking, stiffness, edema, movement limitations, an increased level of pain, and an increased risk of falling, thereby compromising the affected individual’s independence and autonomy and promoting the development of mental disorders and a decreased quality of life [1–3].

Since KOA has multiple characteristics, both morphological and symptomatic, it is diagnosed and its progression is monitored on the basis of positive joint symptoms and radiologic typical findings of the disease [4, 5], and its treatment must be based on a multifactorial approach [1, 2, 6]. The symptoms of KOA and the typical abnormalities that are evident in X-rays are not always interdependent. Thus, according to previous studies in the literature, KOA is diagnosed on the basis of radiological severity as well as instruments that screen patients for the symptoms of the disease, which are indicative of symptomatic OA [4, 5].

According to Felson [7], the more severe the radiological grade, as assessed by the Kellgren-Lawrence (K&L) scale, the greater the probability is that the patient presents with knee OA symptoms. This scale grades radiological severity on a scale from 0 to 4 on the basis of changes in the joint, such as the appearance of osteophytes, the narrowing of the joint space, sclerosis subchondral bone, and deformities [8]. In addition, higher radiological grades on the K&L scale are associated with the presence of depressive symptoms [9], more severe pain, more severe stiffness, poorer functional difficulty scores [10–12], and worse performance in functional tests predictive of fall risk [12, 13]. Furthermore, the presence of mild cognitive impairment is associated with a higher incidence of KOA [14].

However, it has also been documented that the presence of depressive symptoms, pain, functional difficulty, and a risk of falls are not associated with radiological severity [15–19], and the relationship between radiological severity and cognitive loss in elderly people with KOA has not been explored sufficiently.

Therefore, it is not clear how the radiological severity determined by the Kellgren-Lawrence scale are related to the scores of instruments that measure mental health (depressive symptoms and cognitive loss), quality of life related to KOA (pain, stiffness and functional difficulty), and functionality (risk of falls), such as the Geriatric Depression Scale, the Mini-Mental State Examination, the WOMAC questionnaire, the timed up and go test, and the Berg balance scale.

Thus, this study aimed to investigate the relationship between radiological severity, as assessed by the individual grades and grouped grades (grades “0 and 1” and “2 to 4”) of the Kellgren-Lawrence scale (K&Ls), and depression symptoms, cognitive loss, risk of falls, and quality of life in relation to knee osteoarthritis, as assessed by other instruments.

## Materials and methods

The data in this study were retrieved from an original database used in the following study, which was carried out in 2013–2014 in the municipality of Amparo in São Paulo, Brazil: “Comparative analysis of the epidemiological profile of the elderly in a community: a Cohort Study”. It included individuals who were registered in the Family Health Program (FHP-Amparo) and met the inclusion and exclusion criteria.

The inclusion criteria were as follows: 60 years of age or older, a permanent address in the city, radiologic examination severity indicative of KOA, and data for all instruments included in this study. The exclusion criteria were as follows: not having understood any of the questions or tests included in the evaluation protocols; having undergone knee joint reconstruction surgery; and having physical or mental impairment, severe visual, auditory or cognitive impairment, or an incapacitating disease that prevented the completion of the proposed evaluations, such as Parkinson’s disease and neoplasia in the terminal stage.

### Assessment instruments

This project was approved by the local research ethics committee under number 05622818.6.0000.5404. The outcome measures were the individual and grouped grades of radiological severity and the scores of instruments assessing depressive symptoms, cognitive loss, risk of falls, and quality of life (specifically for osteoarthritis). All questionnaires, tests, and exams were administered by specialists, including trained health agents, interviewers, and doctors, during a single visit to the home of the elderly participant or to the PSF Amparo health unit.

To classify cases of knee osteoarthritis, we used the Kellgren-Lawrence scale, which grades changes detected on a radiograph on a scale from 0 to 4 (grade 0—normal: no joint changes; grade 1—suggestive: the findings are suggestive of the presence of osteophytes; grade 2—mild: the presence of small osteophytes and possible narrowing of the joint space; grade 3—moderate: the presence of a large quantity of osteophytes that are moderate in size, narrowing of the joint space and possible deformation of the bony extremities; grade 4—severe: the presence of a large quantity of osteophytes that are large in size, severe narrowing of the space joint, subchondral bone sclerosis, and bone deformity) [8]. In the present study, all radiographs were evaluated by two doctors, a radiologist and a rheumatologist independently, and they were blinded to the patients’ clinical variables, increasing the reliability of the radiological evaluation. The intraobserver and interobserver reliability was assessed using the Kappa coefficient of agreement.

The mental health of the elderly individuals was investigated by the Geriatric Depression Scale (GDS) and Mini-Mental State Examination (MMSE) instruments, which are used to assess patients’ psychological conditions [20] and to identify cognitive loss [21, 22], respectively. For a subjective assessment of the quality of life of people with osteoarthritis, the WOMAC questionnaire (Western Ontario and McMaster Universities Osteoarthritis Index) was used, and three domains were assessed: pain (five items), stiffness (two items), and physical activity (17 items). The sum of the scores for the domains constituted the WOMAC score [23, 24]. To determine patients’ risk of falling on the basis of their physical condition, their mobility and balance were assessed using the timed up and go test (TUG) [25, 26] and the Berg balance scale (BBS) [27, 28].

### Statistical analysis

The characteristics of the study population were assessed according to the variables studied, and the categorical variables are expressed in a table as the absolute frequency (*n*) and percentage (%) values. The descriptive statistics of the continuous variables are expressed as the means, standard deviations, minimum and maximum values, medians, and quartiles. To compare the categorical variables between the radiologic grades determined by the Kellgren-Lawrence scale, Fisher’s exact test was used for variables with fewer than five categories.

Because the data were not normally distributed, the Mann-Whitney test for two categories and the Kruskal-Wallis test for three or more categories were used to assess the continuous variables according to the osteoarthritis grades. To analyze the relationships between the continuous variables and osteoarthritis grades, Spearman’s correlation coefficient was calculated. A significance level of 5% (*p* < 0.05) was used, and SAS for Windows, version 9.2, was used for statistical analysis (SAS Institute Inc., 2002–2008, Cary, NC, USA).

## Results

A total of 181 subjects were selected to participate in this study; the mean age was 67.3 ± 6.8 years, 56.35% were female, and 43.64% were male. The radiological severity is described in Table 1. A total of 53.57% of the females were included in the grades 0 and 1 group, and 60.86% were included in the grades 2 to 4 group.

**Table 1:** Description of the sample by radiological severity (K&L)

Radiological grade	0	1	2	3	4
Number of elderly	60	52	24	32	13
Frequency (%)	33.15	28.73	13.26	17.68	7.18

When the K&L scale grades were divided into two groups, classified by the presence (grades 2 to 4) or absence (grades 0 and 1) of knee osteoarthritis (KOA), 69 and 112 subjects were included in the grades 2 to 4 and grades 0 and 1 groups, accounting for 38.12% and 61.88% of the study population, respectively. Most of the population evaluated in this study had radiological grades that were not indicative of the presence of KOA. A minority of the population was diagnosed with the disease, with a predominance of grade 3 (moderate).

The average scores for the GDS, MMSE, and WOMAC tests indicated that the population was generally had a “mild” level of depressive symptoms and cognitive loss and a “low” level of pain, stiffness, and functional difficulty. The average scores in the TUG and BBS tests indicated that the elderly people had a low risk of falling (Table 2).

**Table 2:** Comparison between grades of the Kellgren-Lawrence between categorical variables and description of the sample regarding the performance in the evaluated tests

Variable	Grade 0	Grade 1	Grade 2	Grade 3	Grade 4	*p* value*	Mean (SD)
	*N* (%)	*N* (%)	*N* (%)	*N* (%)	*N* (%)		
GDS						*p* = 0.988	6.89 (1.68)
0–5	9 (16.36)	5 (12.50)	6 (18.75)	8 (20.00)	2 (15.38)		
6–10	43 (78.18)	35 (85.00)	25 (78.13)	30 (75.00)	11 (84.62)		
11–15	3 (5.45)	1 (2.50)	1 (3.13)	2 (5.00)	0 (0.00)		
MMSE						*p* = 0.774	23.45 (3.98)
0–9	1 (1.82)	0 (0.00)	0 (0.00)	0 (0.00)	0 (0.00)		
10–20	9 (16.36)	8 (20.00)	7 (21.88)	12 (30.00)	3 (23.08)		
21–26	28 (50.51)	22 (55.00)	13 (46.88)	22 (33.00)	6 (46.15)		
≥ 27	17 (30.91)	10 (25.00)	10 (31.23)	6 (15.00)	4 (30.77)		
WOMAC						*p* = 0.450	16.25 (21.4)
0–25	19 (31.67)	18 (34.62)	8 (33.33)	5 (15.63)	2 (15.38)		
26–50	29 (48.33)	24 (46.15)	12 (50.00)	18 (56.23)	4 (30.77)		
51–75	8 (13.33)	6 (11.54)	2 (8.33)	5 (15.63)	4 (30.77)		
76–100	4 (6.67)	4 (7.69)	2 (8.33)	4 (12.50)	3 (23.08)		
TUG						*p* = 0.124	10.88 (4.74)
0–10	32 (53.33)	28 (53.85)	15 (62.50)	19 (59.30)	5 (38.46)		
11–20	27 (45.00)	24 (46.15)	9 (37.50)	11 (34.38)	6 (46.15)		
21–29	1 (1.67)	0 (0.00)	0 (0.00)	0 (0.00)	2 (15.38)		
≥ 30	0 (0.00)	0 (0.00)	0 (0.00)	2 (6.25)	0 (0.00)		
BBS						*p* = 0.061	50.28 (6.65)
0–36	3 (5.000	1 (1.92)	2 (8.33)	2 (6.25)	3 (23.08)		
37–44	1 (1.67)	2 (3.85)	1 (4.17)	4 (12.50)	1 (7.69)		
45–56	56 (93.33)	49 (94.23)	21 (87.50)	26 (81.25)	9 (69.23)		

*p value for the Fisher’s exact test

### Relationship between the scores of the instruments evaluated and the radiological grades of the Kellgren-Lawrence scale

When each of the grades of the K&L scale, including 0, 1, 2, 3, and 4, was compared to the GDS, MMSE, WOMAC, TUG, and BBS scores, no significant differences were observed in any of the categorical variables (Table 2). The same result was observed in the comparative analysis in relation to the continuous variables (Table 3).

**Table 3:** Comparison between grades of the Kellgren-Lawrence between continuous variables

Variable	Grade 0	Grade 1	Grade 2	Grade 3	Grade 4	*p* value*
	Mean (SD)	Mean (SD)	Mean (SD)	Mean (SD)	Mean (SD)	
GDS	6.98 (1.84)	6.95 (1.50)	6.47 (1.61)	7.08 (1.76)	6.77 (1.48)	*p* = 0.314
MEEM	23.87 (4.06)	23.63 (3.56)	24.13 (4.32)	22.43 (3.63)	22.62 (4.87)	*p* = 0.205
WOMAC	13.60 (19.07)	13.23 (18.58)	15.08 (19.70)	19.69 (24.17)	34.23 (30.06)	*p* = 0.064
TUG	10.28 (3.31)	10.21 (2.27)	10.32 (2.46)	12.16 (8.53)	14.19 (6.38)	*p* = 0.265
BBS	50.92 (5.86)	51.37 (6.12)	50.54 (6.23)	48.66 (7.29)	46.46 (9.70)	*p* = 0.179

*p value for the Kruskal-Wallis test

The results suggest that when the radiological grades are independently evaluated, the K&L scale is not statistically related to any of the tests evaluated.

### Relationship between the scores of the instruments evaluated and the radiological grades of the Kellgren-Lawrence scale divided into two groups

When the grades of the K&L scale classified into two groups, “0 and 1” and “2 to 4”, were compared with the categorical variables, the BBS score significantly differed by the K&L grade, with a higher frequency of high and moderate grades, from 2 to 4, with higher BBS scores (*p* = 0.031, Additional file 1). This result suggests that elderly people with KOA more commonly have a moderate or high risk of falling. For the other instruments evaluated, no significant differences were found (Additional file 1).

The comparison between groups and continuous variables showed a significant difference in the WOMAC and BBS scores (Table 4). These results indicate that the levels of pain, stiffness, functional difficulty, and risk of falling are higher in elderly people with KOA than in elderly people without KOA.

**Table 4:** Comparative analysis between groups and continuous variables

Variable	Group 0 and 1 (*N* = 112)	Group 2 to 4 (*N* = 69)	*p* value*
	Mean (SD)	Mean (SD)	
GDS	6.97 (1.70)	6.80 (1.67)	*p* = 0.298
MMSE	23.77 (3.84)	23.09 (4.13)	*p* = 0.295
WOMAC	13.43 (18.76)	20.83 (24.57)	***p*** **= 0.026**
TUG	10.25 (2.86)	11.90 (6.65)	*p* = 0.408
BBS	51.13 (5.96)	48.90 (7.49)	***p*** **= 0.035**

*p value for the Mann-Whitney test

### Correlation between continuous variables and the grades of the Kellgren-Lawrence scale

In the correlation analysis between the continuous variables and grades of KOA (in the total study population and subgroups), a significant correlation was found for the WOMAC score (Table 5).

**Table 5:** Analysis of correlation between continuous variables and the grades of the Kellgren-Lawrence

Variable	Total Sample (*N* = 181)		Group 0 and 1 (*N* = 112)		Group 2 to 4 (*N* = 69)	
	*r*	*p*	*r*	*p*	*r*	*p*
GDS	− 0.03544	0.6367	− 0.01559	0.8808	0.16215	0.1382
MEEM	− 0.01185	0.8746	− 0.07265	0.4841	0.05039	0.6470
WOMAC	0.17099	**0.0214**	− 0.02285	0.8110	0.22709	0.0606
TUG	0.08274	0.2681	0.00831	0.9307	0.20181	0.0963
BBS	− 0.14007	0.0600	0.04691	0.6234	− 0.15289	0.2098

*r* Spearman’s correlation coefficient, *p p* value, *N* number of subjects

This result suggests that the higher the radiological grade of KOA, the higher the WOMAC score is.

### Comparative and correlation analyses between the Kellgren-Lawrence grades and WOMAC domain scores

Based on the results presented, significant differences between the WOMAC domains and the Kellgren-Lawrence scale were suspected, and the differences were investigated. The three WOMAC domains (pain, stiffness, and functional limitations) were compared with the individual grades and groups of grades (0 and 1 and 2 to 4) (Additional files 2 and 3). In addition, a correlation analysis was performed between the WOMAC domain scores and the radiological scores in the total study population and in subgroups (Additional file 4). No significant differences were observed in any of the analyses.

## Discussion

The aim of this study was to evaluate the relationship between radiological severity, as assessed by the individual and grouped grades (“0 and 1” and “2 to 4”) of the Kellgren-Lawrence scale, and mental and physical health in seniors, as assessed by other instruments. This study is the first to compare the individual grades of the K&L scale and factors associated with KOA, such as depressive symptoms, cognitive loss, quality of life in relation to OA, and risk of falling in elderly Brazilians. In addition, all these factors associated with KOA were assessed in the same study.

When they were assessed independently, the grades of the K&L scale were not related to the score of any of the investigated instruments. When the K&L grades were assessed in groups on the basis of the presence or absence of KOA (groups 2 to 4 and 0 and 1) in relation to the results of the GDS and MMSE tests, there were no differences between individuals with or without KOA. However, we should consider that most of the participants included in the study had depressive symptoms and mild cognitive loss, and because this is a cross-sectional study, we cannot state that there is no relationship between the K&L scale and these questionnaires.

According to El Monaem et al. [9], the K&L radiologic classifications are correlated with the emergence of depression in individuals with KOA, as assessed by the Beck depression inventory (BDI). However, that study used another questionnaire, suggesting that there may be differences between these assessment instruments (GDS and BDI); moreover, according to the findings of Bentz and Hall [29], the GDS has a greater ability to correctly diagnose depression than does the BDI.

Regarding cognitive loss and its association with K&L grades, our results differed from the findings reported by Yoshimura et al. [14], which suggested there are significantly lower MMSE scores in individuals with radiological KOA (grade ≥ 2) than is those without radiological KOA (grade 0 and 1). In addition, the results suggest that the incidence of radiological OA decreases as the MMSE score increases. However, the authors did not investigate only elderly people and aimed to investigate whether cognitive loss increases the risk of KOA, without specifically evaluating the relationship between the severity of radiological severity and the presence of cognitive loss.

When we assessed the radiological severity in groups, we found that the high and moderate BBS scores, indicating a high risk of falling, were more frequent in individuals with radiological OA that in those without radiological OA. In addition, in the WOMAC and BBS tests, the mean values indicated that the participants had both a low WOMAC score and low BBS score, indicating a low risk of falling. Nevertheless, the difference in the mean between groups suggests that group “2 to 4” had poorer conditions that did the group “0 and 1,” with higher WOMAC scores and lower BBS scores. In this sense, it is understood that even when they are in good health regarding the level of pain, stiffness, functional difficulty, and risk of falling, elderly people with radiological OA (grades 2 to 4) have more impairments than do those without OA (grades 0 and 1).

Kim et al. [13] indicated that patients with moderate and severe knee OA (grades 3 and 4) have worse functional performance according to the BBS than do those with mild OA (grade 2 or less), which is similar to our results. On the other hand, it has been documented that individuals with a worse K&L classification also perform worse on the TUG test [12, 13], which was not observed in our study. Our results are similar to those of Kumar [19], who evaluated the knee joint and showed that the TUG results and the K&L classifications, when evaluated in groups, are not related. The inconsistency between the results of these instruments can be justified by the differences in the study population evaluated, few participants were classified as having high and moderate risk levels according to the TUG due to the different physical tasks that each test evaluates. However, it was expected that this population would have difficulty performing these tests due to the presence of morphological change that hinders joint functionality.

Regarding our results, a significant relationship between the WOMAC score and the radiological grade in individuals with KOA has been documented [12]. A worsening WOMAC score and pain score is more suggestive of radiological grades 3 and 4 than grades 0 to 2. Radiological changes resulting from KOA are significantly associated with the presence of pain [30]. A large proportion of individuals with radiological grades 3 and 4 have recurrent pain. In addition, the proportion of those with pain increases with the severity of radiological changes, as assessed by the K&L scale [30]. According to Pereira et al. [31], higher levels of pain are reported by individuals classified as having grades ≥ 2 on the K&L scale. This finding justifies why an instrument that measures pain, such as the WOMAC, is related to imaging findings.

On the other hand, according to Creamer et al. [15], the changes in the KOA joint and the functional difficulty, as measured by the WOMAC, are not correlated, and according to Kumar et al. [19], the K&L classifications in groups with OA (notes 2 and 3) and without OA (notes 0 and 1) are not related to pain or joint function. However, Kumar et al. [19] investigated the hip joint and used another assessment tool, which may explain the inconsistency in the results found. According to Szebenyi et al. [11], the likelihood of individuals with KOA presenting with pain or reduced function, as assessed by the WOMAC, is greater if these changes occur in the tibiofemoral (medial and/or lateral) and patellofemoral compartments at the same time rather than in one of these locations only, which was not assessed by Creamer et al. [15]. In addition, pain was more strongly associated with specific changes caused by KOA [11, 16, 32–34]. Therefore, it seems that the type of change in the K&L scale is more effective in predicting pain and functional decline than the grade itself.

In the correlation analysis, the results indicated the WOMAC score is significantly associated with the radiological grade in the total study population. This result shows that pain, stiffness, and functional difficulty worsen as the radiological grade increases. This finding corroborates the findings of Szebenyi et al. [11], who also suggested that there is a correlation between pain and function and the K&L grade. In addition, according to Kim et al. [10], there is a significant correlation between the K&L classification and the total score and all subscales of the WOMAC. In contradiction to this finding regarding the WOMAC domains, our results showed no significant differences or correlations between the three domains and the grades, assessed in groups, in the total study population [18, 35]. In contrast to our findings, those of other studies have suggested that the WOMAC score and the K&L radiological grades are not correlated [16, 18]; unlike in our study, most of the study populations in these studies were considered to have a K&L grade of 3.

Regarding limitations, this study included a cross-sectional analysis, a cohort selected by convenience sampling, and a small number of individuals in each group established by the radiological severity and each subgroup established by the assessment instrument scores, as well as a small number of individuals diagnosed with KOA. Additional studies are recommended to better understand the relationship between mental and physical health and the radiological classifications determined by the K&L scale.

## Conclusion

The radiological grades of the Kellgren-Lawrence scale, when evaluated independently, were not related to the scores of the GDS, MMSE, WOMAC, TUG, and BBS assessment instruments. However, when the scores were evaluated in groups, individuals with established radiological KOA had poorer WOMAC and BBS scores than did the individuals with none or few radiological features in plain X-rays. In addition, the WOMAC total score was positively correlated with the Kellgren-Lawrence grade but not with depressive symptoms, cognitive loss, or risk of falling.

## Supplementary information

**Additional file 1.** Comparative analysis between groups and categorical variables.

**Additional file 2.** Comparative analysis between the WOMAC domains and the grades of the Kellgre-Lawrence.

**Additional file 3.** Comparative analysis between the WOMAC domains and the groups of Kellgren-Lawrence grades.

**Additional file 4.** Analysis of correlation between the WOMAC domains and the grades of the Kellgren-Lawrence.

## Data Availability

The datasets used and/or analyzed during the current study are available from the corresponding author on reasonable request.

## Abbreviations

K&L: Kellgren-Lawrence
GDS: Geriatric Depression Scale
MMSE: Mini-Mental State Examination
WOMAC: Western Ontario and McMaster Universities Osteoarthritis Index
TUG: Timed up and go test
BBS: Berg balance scale
KOA: Knee osteoarthritis
FHP: Family Health Program
