# Supplementary material for: Relationship between radiological severity and physical and mental health in elderly individuals with knee osteoarthritis
Source: Arthritis Res Ther. 2020 Aug 12;22:187. doi: 10.1186/s13075-020-02280-2 (PMC7425047; doi:10.1186/s13075-020-02280-2)
Supplement: Supplementary file 1 — Additional file 1. Comparative analysis between groups and categorical variables. [file 13075_2020_2280_MOESM1_ESM.docx]

**Additional file 1. Comparative analysis between groups and categorical variables.**

|  | Group 0 and 1 | Group 2 to 4 |  |
| --- | --- | --- | --- |
| Variable | N (%) | N (%) | p-Value* |
| GDS |  |  | p=0.751 |
| 0 - 5 | 14 (14.74) | 17 (18.82) |  |
| 6 – 10 | 78 (81.05) | 67 (77.65) |  |
| 11 - 15 | 5 (4.21) | 3 (3.53) |  |
| MMSE |  |  | p=0.435 |
| 0 - 9 | 1 (1.05) | 0 (0.00) |  |
| 10 - 20 | 17 (17.89) | 22 (25.88) |  |
| 21 - 26 | 51 (52.63) | 43 (50.59) |  |
| >=27 | 27 (28.42) | 20 (23.53) |  |
| WOMAC |  |  | p=0.275 |
| 0 - 25 | 37 (33.04) | 15 (21.74) |  |
| 26 - 50 | 53 (47.32) | 34 (49.28) |  |
| 51 - 75 | 14 (12.50) | 11 (15.94) |  |
| 76 - 100 | 8 (7.14) | 9 (13.04) |  |
| TUG |  |  | p=0.169 |
| 0 – 10 | 60 (53.57) | 39 (56.52) |  |
| 11 – 20 | 51 (45.54) | 26 (37.68) |  |
| 21 – 29 | 1 (0.89) | 2 (2.90) |  |
| >=30 | 0 (0.00) | 2 (2.90) |  |
| BBS |  |  | **p=0.031** |
| 0 – 36 | 4 (3.57) | 7 (10.14) |  |
| 37 - 44 | 3 (2.68) | 6 (8.70) |  |
| 45 - 56 | 105 (93.75) | 56 (81.16) |  |

* p-Value: Result related to Fisher's exact test.
