# Supplementary material for: Relationship between radiological severity and physical and mental health in elderly individuals with knee osteoarthritis
Source: Arthritis Res Ther. 2020 Aug 12;22:187. doi: 10.1186/s13075-020-02280-2 (PMC7425047; doi:10.1186/s13075-020-02280-2)
Supplement: Supplementary file 2 — Additional file 2. Comparative analysis between the WOMAC domains and the grades of the Kellgre-Lawrence. [file 13075_2020_2280_MOESM2_ESM.docx]

**Additional file 2. Comparative analysis between the WOMAC domains and the grades of the Kellgre-Lawrence.**

|  | Grade 0 (N=60) | Grade 1 (N=52) | Grade 2 (N=24) | Grade 3 (N=32) | Grade 4 (N=13) |  |
| --- | --- | --- | --- | --- | --- | --- |
| Domains | Mean (SD) | Mean (SD) | Mean (SD) | Mean (SD) | Mean (SD) | p-Value* |
|  |  |  |  |  |  |  |
| PAIN | 5.18 (6.46) | 4.55 (6.07) | 5.76 (6.14) | 4.10 (4.72) | 7.23 (7.98) | p=0.430 |
| STIFFNESS | 1.27 (1.81) | 1.13 (1.83) | 1.30 (1.76) | 1.54 (2.14) | 1.92 (2.60) | p=0.763 |
| FUNCTIONAL LIMITATION | 9.80 (15.03) | 7.45 (12.32) | 9.03 (12.54) | 10.92 (15.16) | 16.31(19.80) | p=0.505 |

* p-Value for the Kruskal-Wallis test.
