# Supplementary material for: Relationship between radiological severity and physical and mental health in elderly individuals with knee osteoarthritis
Source: Arthritis Res Ther. 2020 Aug 12;22:187. doi: 10.1186/s13075-020-02280-2 (PMC7425047; doi:10.1186/s13075-020-02280-2)
Supplement: Supplementary file 3 — Additional file 3. Comparative analysis between the WOMAC domains and the groups of Kellgren-Lawrence grades. [file 13075_2020_2280_MOESM3_ESM.docx]

**Additional file 3. Comparative analysis between the WOMAC domains and the groups of Kellgren-Lawrence grades.**

|  | Group 0 and 1 (N=112) | Group 2 to 4 (N=69) |  |
| --- | --- | --- | --- |
| Domains | Média (DP) | Média (DP) | p-Value* |
| Pain | 4.92 (6.27) | 5.22 (5.90) | p=0.430 |
| Stiffness | 1.21 (1.81) | 1.51 (2.06) | p=0.487 |
| Functional Limitation | 8.81 (14.04) | 11.01 (15.02) | p=0.205 |

* p-Value for the Mann-Whitney test.
