# Supplementary material for: Relationship between radiological severity and physical and mental health in elderly individuals with knee osteoarthritis
Source: Arthritis Res Ther. 2020 Aug 12;22:187. doi: 10.1186/s13075-020-02280-2 (PMC7425047; doi:10.1186/s13075-020-02280-2)
Supplement: Supplementary file 4 — Additional file 4. Analysis of correlation between the WOMAC domains and the grades of the Kellgren-Lawrence. [file 13075_2020_2280_MOESM4_ESM.docx]

**Additional file 4. Analysis of correlation between the WOMAC domains and the grades of the Kellgren-Lawrence.**

|  | Total Sample (N=181) | | Group 0 and 1 (N=112) | | Group 2 to 4 (N=69) | |
| --- | --- | --- | --- | --- | --- | --- |
| Variable | r | p | r | p | r | p |
| Pain | 0.03497 | 0.6412 | -0.06123 | 0.5556 | -0.01137 | 0.9178 |
| Stiffness | 0.03701 | 0.6218 | -0.09256 | 0.3723 | 0.05919 | 0.5905 |
| Functional Limitation | 0.07639 | 0.3081 | -0.06566 | 0.5273 | 0.04961 | 0.6521 |

* r = Spearman's correlation coefficient; p = p-Value; N = number of subjects.
